# Influence of chronic L-DOPA treatment on immune response following allogeneic and xenogeneic graft in a rat model of Parkinson’s disease

**DOI:** 10.1016/j.bbi.2016.11.014

**Published:** 2017-03

**Authors:** Ludivine S. Breger, Korbinian Kienle, Gaynor A. Smith, Stephen B. Dunnett, Emma L. Lane

**Affiliations:** a School of Pharmacy & Pharmaceutical Sciences, Redwood Building, King Edward VII Avenue, CF10 3NB Cardiff, UK; b Brain Repair Group, Cardiff School of Biosciences, Museum Avenue, CF10 3AX Cardiff, UK

**Keywords:** Parkinson’s disease, L-DOPA, Transplant, Stem cell, Dyskinesia, Immune response

## Abstract

Although intrastriatal transplantation of fetal cells for the treatment of Parkinson’s disease had shown encouraging results in initial open-label clinical trials, subsequent double-blind studies reported more debatable outcomes. These studies highlighted the need for greater preclinical analysis of the parameters that may influence the success of cell therapy. While much of this has focused on the cells and location of the transplants, few have attempted to replicate potentially critical patient centered factors. Of particular relevance is that patients will be under continued L-DOPA treatment prior to and following transplantation, and that typically the grafts will not be immunologically compatible with the host. The aim of this study was therefore to determine the effect of chronic L-DOPA administered during different phases of the transplantation process on the survival and function of grafts with differing degrees of immunological compatibility. To that end, unilaterally 6-OHDA lesioned rats received sham surgery, allogeneic or xenogeneic transplants, while being treated with L-DOPA before and/or after transplantation. Irrespective of the L-DOPA treatment, dopaminergic grafts improved function and reduced the onset of L-DOPA induced dyskinesia. Importantly, although L-DOPA administered post transplantation was found to have no detrimental effect on graft survival, it did significantly promote the immune response around xenogeneic transplants, despite the administration of immunosuppressive treatment (cyclosporine). This study is the first to systematically examine the effect of L-DOPA on graft tolerance, which is dependent on the donor-host compatibility. These findings emphasize the importance of using animal models that adequately represent the patient paradigm.

## Highlights

• Evaluation of clinical parameters that could influence the success of cell therapy.
• L-DOPA does not impair rodent graft viability.
• L-DOPA treatment following transplantation can influence the host immune response.
• Cell transplant prior to initiation of L-DOPA treatment limits dyskinesia development.

## 1 Introduction

There has been a longstanding debate regarding the potential toxicity of the mainstay therapy for the neurodegenerative movement disorder Parkinson’s disease (PD), L-DOPA. It has been hypothesized that the drug may impact on the development of the disease by hastening or preventing nigral degeneration in PD patients (Quinn et al., 1986, Diamond et al., 1987, Rajput et al., 1997, Fahn et al., 2004). While studies suggest it is not clinically relevant to disease progression, *in vitro* studies have demonstrated that dopaminergic neurons in culture are vulnerable to the oxidative damage caused by L-DOPA (reviewed by Olanow (2015)). The possibility of toxicity however becomes particularly relevant when looking at curative or cell replacement strategies for the treatment of PD.

Fetal cell transplantation of dopaminergic neurons into the caudate putamen was first trialed in 1989 (Lindvall et al., 1990b). Having shown encouraging results in preclinical studies and open-label clinical trials, US-led double-blind placebo controlled studies failed to demonstrate consistent benefit from the graft (Lindvall et al., 1990a, Kordower et al., 1995, Hauser et al., 1999, Hagell and Brundin, 2001). Furthermore these studies, alongside the retrospective video analysis of the London-Lund-Marburg open label study, blew the field into disarray with the discovery of motor side effects persisting after the withdrawal of L-DOPA, now termed graft-induced dyskinesias (GID) (Hagell et al., 2002, Olanow, 2003). In the search to understand inconsistency in transplant efficacy and the source of the motor side effects, it is important to consider factors that are present in patients but absent in models of transplantation in PD (generally the 6-hydroxydopamine (6-OHDA) lesioned rat). In this context, L-DOPA toxicity may be of greater relevance as, at the early stage when they are transplanted, developing neurons may be vulnerable to the effects of pulsatile dopamine flux. The majority of transplant recipients will have been on L-DOPA medication for some time prior to transplantation and will remain on it for a significant period post transplantation, as the graft matures enough to support effective dopamine function.

Preclinical studies have reported contradictory findings: some researchers have described failure of the graft to thrive under L-DOPA treatment (Yurek et al., 1991, Steece-Collier et al., 2009) while others found no detrimental effect of the treatment on the survival of grafted dopaminergic cells or their functional efficacy (Blunt et al., 1990, Blunt et al., 1991, Blunt et al., 1992, Adams et al., 1994). Nonetheless, the role of L-DOPA administration pre- and post-transplantation has not been investigated experimentally in a systematic manner. Furthermore, most of these papers have used ventral mesencephalon (VM) harvested from the same strain of rat as the hosts, in order to avoid a graft-induced immune response. While simplifying the model, this has again neglected a factor, which is critical when considering the transplantation of patients. To the best of our knowledge, only one paper has thus far combined non-syngeneic VM transplants and L-DOPA treatment (Soderstrom et al., 2008). In that paper, L-DOPA was administered to all groups with the focus being to explore the impact of inflammation on the synaptic reorganization occurring the presence of L-DOPA. The study was however not designed to compare the impact of L-DOPA treatment pre- and post- transplantation in a systematic manner.

In determining the effect of L-DOPA on transplanted fetal dopaminergic precursors it is therefore paramount to apply this technique in an improved simulation of ‘real world’ conditions. The use of syngeneic tissue does not trigger a significant immune response in rodents. Patients receive pooled allogeneic tissue from several donors and post-mortem analysis performed on transplanted patients has illustrated that, even in well surviving grafts, there are infiltrating B- and T-lymphocytes in the grafted putamen indicative of an inflammatory host response in the graft (Kordower et al., 1997). Human genetic diversity is such that, an allograft paradigm is insufficiently aggressive to model the immunogenicity that would be associated with transplanting pooled tissue coming from multiple donors, as is the case in fetal transplantation for Parkinson’s disease. Consequently using a donor from a closely related species (e.g. mouse into rat), termed a concordant xenograft, reproduces many aspects of the humoral response observed in transplants for MHC-mismatched allotransplants and would be a more appropriate animal model (Sanchez-Mazas, 2007). This idea has already been used in primates in whom concordant xenografts are used to model human allotransplantation (Michler et al., 1996). This provides a more representative model of the pooled unmatched allograft used to treat PD, than those models used previously. The present study was therefore designed to specifically address the hypothesis that L-DOPA may impact upon graft survival and function in an immunologically incompatible graft. This paradigm is as akin to transplantation conditions in patients as possible, assessing the survival and function of allogeneic and xenogeneic VM grafts while subject to different L-DOPA administration regimes.

## 2 Materials and methods

### 2.1 Experimental design

**Fig. 1:**
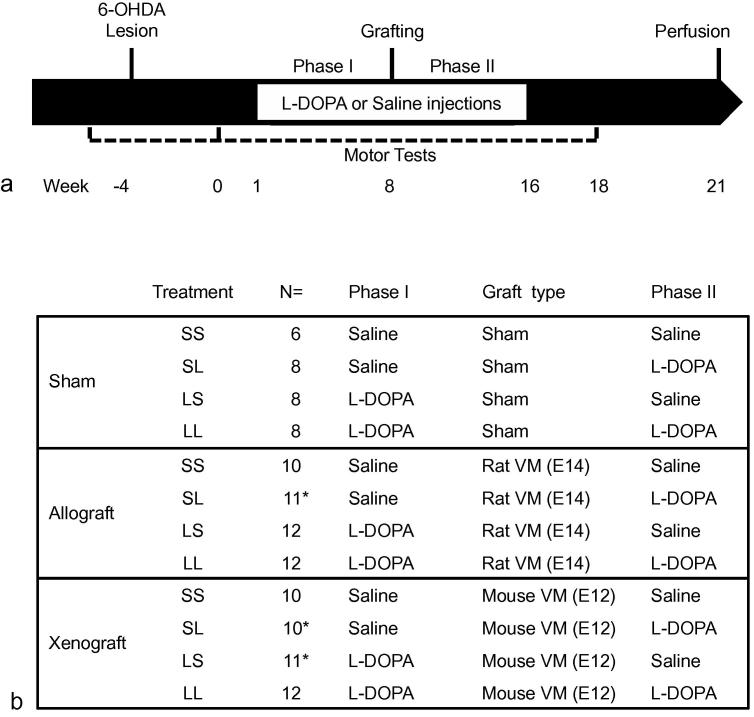
Experimental design. (a) Timeline of the study. (b) Treatment and transplantation received by the animals for each group. The group were named after the treatment they received in treatment phase 1 and 2: S = saline, L = L-DOPA thus, SS group received saline in treatment phase 1 and 2, SL group received saline in treatment phase 1 and L-DOPA in treatment phase 2, LS received L-DOPA in Treatment phase 1 and saline in treatment phase 2 and LL received L-DOPA in treatment phase 1 and 2. ^*^These groups originally numbered 12 animals but some developed tumors unrelated to the experiment, so had be removed from the study.

122 female Sprague Dawley rats were unilaterally lesioned using 6-OHDA HBr and split into balanced experimental groups based on the severity of motor impairment following lesion as evaluated by amphetamine-induced rotations, cylinder, vibrissae and stepping tests. The animals were chronically treated with saline or L-DOPA for 8 weeks (phase I: daily for 4 weeks then every other day) before receiving either: an allograft, a xenograft or sham surgery. Animals receiving xenotransplants were treated daily with cyclosporine A (CSA) to avoid rapid rejection of the graft and better mimic the human conditions. In contrast, allografted animals did not receive any immunosuppressive treatment, as allografts are usually well tolerated in rats. Moreover, this would allow a full immunological response to be observed, should the presence of L-DOPA have an impact. All animals were treated again either with L-DOPA or saline for another 8 weeks following grafting (Fig. 1, phase II: daily for 4 weeks then every other day). Dyskinesias were assessed once a week during the treatment phases I and II, both ‘on’ and ‘off’ L-DOPA (Scale B, as described in Breger and colleagues (2013)). After 2 weeks to allow complete washout of the drug, motor test were repeated. At the end of the experiment all animals were transcardially perfused with 1.5% paraformaldehyde (Torres et al., 2006).

To verify that there was no overt behavioral consequence of, or interaction between chronic CSA treatment on L-DOPA-induced dyskinesia, a supplementary experiment was carried out. 16 female Sprague Dawley received unilateral 6-OHDA lesions and were selected based on the results of the amphetamine-induced rotation score. They were then separated in 2 groups: 1) treated daily with L-DOPA and CSA (n = 8), 2) treated daily with L-DOPA only (n = 8). Abnormal involuntary movements (AIMs) were scored twice a week, for 4 weeks (L-DOPA 6 mg/kg) and then once a week, for 2 weeks (L-DOPA 12 mg/kg), rotational behavior of the rats was recorded concomitantly.

### 2.2 Animals and materials

Sprague Dawley rats (experiment 1: n = 122 and experiment 2: n = 16; Harlan, UK) weighing 200–220 g at the start of the experiment were housed 2–4 per cages with *ad libitum* access to food and water. The experiments were approved by the Cardiff University AWERB and carried out in accordance with the UK guidelines for the care and use of experimental animals under Home Office License No 30/3036 and European Communities Council Directive (2010/63/EEC). 6-OHDA, L-DOPA and benserazide were obtained from Sigma Aldrich, UK; cyclosporine (250 mg/5 ml) was obtained from Sandoz Pharmaceuticals, UK. Four animals were excluded from the first experiment due to poor heath, unrelated to the experimental treatments.

### 2.3 Surgery

#### 2.3.1 6-Hydroxydopamine lesion

6-OHDA was used to create hemi Parkinsonism in rats. The toxin was delivered directly into the right medial forebrain bundle (MFB), which contains the dopaminergic nigrostriatal pathway. Selective targeting of this site allows near total depletion of nigrostriatal dopaminergic neurons as described by Torres et al. (2011). Briefly, the rats were anaesthetized with 2–3% Isoflurane (IVAK, UK) in a 2:1 O2/NO_2_ mix and received an infusion of 3 μl of a 30 mM solution of 6-OHDA.HBr (Sigma, UK) containing 0.03% acid ascorbic into the right nigrostriatal pathway at the following coordinates AP −4.0 mm from bregma; ML +1.3 mm from midline; DV −7.0 mm below dura, with the nose-bar set at −4.5 mm below the interaural line. The cannula remained in place for 3 min following injection. Postoperatively, animals were sutured and received a 5 ml injection s.c. of 0.9% sodium chloride containing 4% glucose for hydration and 10 μl of analgesic (5 mg/ml Meloxicam, Boehringer Ingleheim, Germany). Post-mortem analysis confirmed that all animals had greater than 95% loss of tyrosine hydroxylase (TH) positive cells in the right substantia nigra (no significant difference was observed between the different group F_11,106_ = 1.01, p = 0.4439, data not shown).

#### 2.3.2 VM transplant

According to a previous study, the optimal embryonic stage for mouse VM transplantation is 12 days post-copulatory (Torres et al., 2007), corresponding to Carnegie stage 16, similarly achieved at 14 days post-copulation in rats (Butler and Juurlink, 1987). Time-mated pregnant female rats (for allograft: E14 Wistar rats) or mice (for xenograft: E12 CD1 mice) were obtained commercially (Harlan, UK) and terminally anesthetized by intra-peritoneal (i.p.) injection of 150 mg/kg Pentobarbital (Merial, UK). The fetal brains were extracted after decapitation and VM were dissected in Hanks’ balanced salt solution (HBSS, Invitrogen, UK). The dissected pieces were prepared using a common dissociation protocol (Bjorklund et al., 1983, Torres et al., 2007). Briefly, VM were pooled in trypsin solution: 0.1% trypsin and 0.05% DNAse (Sigma, UK) in Dulbecco’s minimum Eagle medium (DMEM, Invitrogen, UK) at 37° C for 10 min, then incubated an extra 10 min in trypsin inhibitors (Sigma, UK). After a wash in DNAse 0.1% in DMEM medium, VM were broken into small cells clusters by mechanic dissociation using gentle pipette trituration. The solution was centrifuged at 2000 rpm/min for 3 min and the number of cells was counted using a haemocytometer slide. After centrifugation, the pellet was suspended in an adequate volume of DMEM 0.1% DNAse to obtain a concentration of 100,000 cell/μl and loaded into a 10 μl Hamilton syringe. 2 μl of the cell suspension were injected over 2 min into the dopamine depleted striatum of anaesthetized animals, at the following coordinates: AP: +0.5 mm; ML: −2.5 mm; DV: −5.0 mm and −4.0 mm (1 μl at each depth) with the nose-bar set at −4.5 mm allowing a flat head. The syringe was left in place for an additional 3 min for diffusion before being retracted. Animals transplanted with xenogeneic tissue received cyclosporine A injections (50 mg/kg i.p.) daily from the day preceding surgery as immunosuppressant treatment.

### 2.4 Behavior tests

#### 2.4.1 Amphetamine-induced rotations

The extent of nigrostriatal dopamine depletion induced by the 6-OHDA lesion was evaluated 4 weeks post-surgery, based on the net ipsilateral rotational behavior induced by administration of *d*-Amphetamine (2.5 mg/kg in 0.9% saline i.p., Sigma, UK). The animals were placed in Perspex bowls and harnessed to automated rotometers based on the design of Ungerstedt and Arbuthnott (1970) and the net rotations were recorded over 90 min. Rats were considered adequately lesioned and used for further study if they performed at least the equivalent of an average of 6 full turns per minute (Torres et al., 2011). The same process was used post-transplantation in order to monitor the functional efficacy of the graft.

#### 2.4.2 Vibrissae test

The rats were held in a way that allowed the forelimbs to move freely when the torso and hind limbs were supported and moved upward from under the bench, allowing the whisker on the tested side to gently brush the edge of the table. This should induce an automatic limb placing response on the test side of an intact animal (Fleming and Schallert, 2011). This test was performed 10 times on each side in order to obtain a percentage estimate of response on each side. At each time point (before lesion, after lesion and after grafting) the whole test was repeated 3 times for each animal and the mean number of accurate placements across all tests was calculated.

#### 2.4.3 Stepping test

This task evaluates the ability of the rat to adjust a weight bearing forepaw in response to movement along a flat surface. The rats were held above a flat bench allowing only one forelimb to touch the surface of the table bearing weight (the body weight was largely supported by the experimenter but some weight given to the paw). The rat was moved laterally in both forehand and backhand directions across 1 m of bench over a period of 10 s and the number of adjusting steps made by the paw was counted for each forelimb when moved. This test was repeated 3 times at each time point (before lesion, after lesion, after grafting).

#### 2.4.4 Cylinder test

The rats were placed in a Perspex cylinder (height: 33.5 cm, diameter: 19 cm) and video recorded. The cylinder was placed between two mirrors to allow scoring when the rats were back to the camera. The number of forelimb touches made by each paw, out of the first 20 touches performed on the cylinder wall, was counted. Performance in the cylinder test was used to assess the extent of the lesion and split the animals evenly amount the groups before treatment and surgery. All of the above behavior tests were carried out by an experimenter blind to the treatment of the animals.

#### 2.4.5 Dyskinesia and stereotypic behavior

L-3,4-Dihydroxyphenylalanine (L-DOPA) methyl ester HCl 10 mg/kg and 15 mg/kg of benserazide HCl, dissolved in 0.9% saline was administered i.p. daily. Dyskinesias were assessed once a week, both in the presence and absence of L-DOPA treatment using an AIM rating scale (scale B) and a stereotypic rating scale as described in a previous study (Breger et al., 2013).

### 2.5 Immunohistochemistry

Upon completion of all behavioral tests, all animals were killed under general barbiturate anesthesia (Pentobarbital, 150 mg/kg i.p.) by transcardial perfusion with 0.2 M buffered saline followed by 1.5% paraformaldehyde (PFA), the brains removed, and post fixed in PFA and immersed in 20% buffered sucrose until they sank (this protocol has been refined and validated in our laboratory see Torres et al., 2006). The brains were then cut on a freezing sledge microtome at 40 μm and sections collected in 12 parallel series. 3,3′-Diaminobenzidine tetrahydrochloride hydrate (DAB, Sigma, UK) immunohistochemistry staining was performed as previously described (Torres et al., 2007), using the following antibodies anti-TH (AB152, Millipore, 1:2000); anti-CD45, anti-CD4 and anti-CD8 (MCA48GA, AbD Serotec, 1:500); anti-Ox42 (MCA275G, AbD Serotec, 1:2000), anti-CD68 (MCA341R, AbD Serotec, 1:100), anti-glial fibrillary acidic protein (GFAP) (*Z*0334, Dako, 1:1000), anti-interleukin-1 beta (IL1β), anti-tumor necrosis factor (TNFα), anti-interferon gamma (IFNγ) (Neoscientific, A1112, A0242, A11534, 1:200), anti-rabbit IgG (BA-1000, Millipore, 1:200), anti-mouse IgG (BA-2001, Vector, 1:200). The slides were allowed to dry at room temperature over night before being dehydrated and delipidated in a succession of ethanol and xylene solutions. They were then cover-slipped with distyrene plasticizer and xylene (DPX). The experimenter was blind to the experimental groups in assessment of the histology. TH^+^ cell bodies were counted on a Leica light microscope (20×) for all sections from a 1 in 12 series through the rat striatum and the total number of dopaminergic cells was estimated using the Abercrombie method (Hedreen, 1998). TH + fiber outgrowth was measured by counting the number of intersection of fibers on a 10 × 10 square grid (each square 500 μm^2^; magnification: 20×) located immediately at the graft host border (periphery) or in the center of the graft (center). Ox42 cells were counted within 3 frames (572 × 429 μm; magnification: 20×), one placed in the middle of the graft and two at the transplant-host border. Leukocyte counts were carried out using an automated stereology microscope (Olympus BX50) and a PC-based image analysis software (Olympus C.A.S.T grid system version 1.6). The whole striatum was outlined (4×) and the enclosed area was measured by the software. Sections within the selected striatal area were sampled randomly and CD45 + cells were counted within a regular series of 286 × 214 μm window (40×). The total number of cells was estimated using the following formula: N = Σ{n × (A/a)} × F × T/(T + H) where n = number of cells counted, A = inclusion area (striatum), a = total sampling area, F = frequency of the sections (×12), T = thickness of the sections (40 μm) and H = mean diameter of the cells. The surface area of the blood vessel was estimated by taking pictures of the sections, stained for Reca-1 (×10), on a Leica DMRBE microscope and analyzing them with ImageJ software (version 1.45, National Institutes of Health, USA). The pictures were converted in 8-bit black and white pictures and the threshold was adjusted to avoid background noise, before the stained area was measured by the software and used as a measure of blood vessel area.

### 2.6 Statistical analysis

Although the nature of dyskinesia rating data is ordinal, non-parametric tests do not permit the analysis of interactions between the different key factors of experimental interest (Times, Type of treatment, Immunological transplantation barriers) as is required for the factorial design used in all experiments. Therefore, since 1) the categories of dyskinesia rating are strictly interval and monotonic 2) inspection of the data indicated that the scores were well distributed between the different categories and 3) analysis of variance is recognized to be extremely robust to derivation of the data from the normality of distribution, that is the basis of the underlying mathematics (Box, 1953), parametric two- and three-factor split plot ANOVA was used for all analyses, with Newman-Keuls and Sidak’s post hoc tests as appropriate to determine the locus of specific significant effects.

## 3 Results

### 3.1 L-DOPA treatment does not affect the survival or the function of the graft

**Fig. 2:**
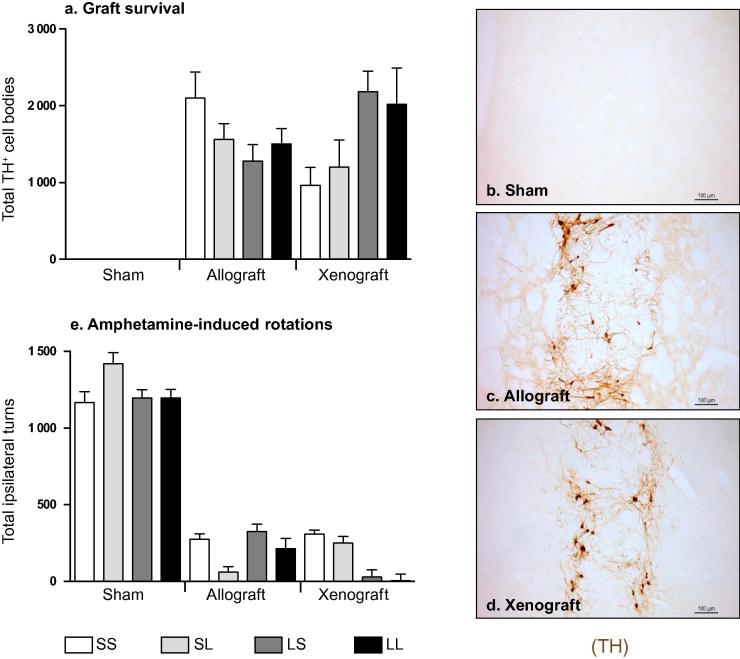
Graft survival and function. (a) The data represent the total number of tyrosine hydroxylase (TH) positive cell bodies present in the transplanted striatum. (b–d) Pictures are representative of each type of graft (these 3 pictures have been selected from the L-DOPA naïve group SS but all the grafts looked similar within each transplant group). (e) Amphetamine-induced rotational behavior. The data represent the relative number of ipsilateral turns performed over 90 min, 10 weeks after transplantation. All data are presented as mean ± SEM for each group, total n = 118.

Graft survival was determined by counting dopaminergic TH positive cell bodies present in the striatum. Both allogeneic and xenogeneic transplanted cells survived well (1675 ± 133 and 1603 ± 193 average of TH positive cell bodies respectively). These data are comparable to previous study that reported a survival of syngeneic transplanted VM ranging between 0.7% and 1.5% (Steece-Collier et al., 2009, Heuer et al., 2013, Tamburrino et al., 2015). There was no significant difference between the numbers of TH positive cell bodies in the allogeneic versus xenogeneic transplanted groups (Fig.2a–d; F_2,115_ = 34.33; Sidak’s post hoc test p = 0.999). The allogeneic transplant seems to better innervate the host striatum than the xenograft, however, the difference was not significant. Moreover, L-DOPA treatment had no impact on the innervation (mean graft to host fiber density allograft SS: 52.9 ± 9.0; allograft LL: 64.33 ± 8.6; xenograft SS: 35.0 ± 6.0; xenograft LL: 34.36 ± 5.3). Similarly, L-DOPA regime (SS, SL, LS, LL) had no impact on the survival of transplanted cells (Fig.2a–d; F_3,106_ = 0.979; p = 0.406), suggesting that chronic treatment with L-DOPA pre and/or post graft did not affect the survival of dopaminergic neurons, regardless of immunogenic compatibility.

The functionality of the transplant was assessed 10 weeks post-transplantation by recording the total number of amphetamine-induced rotations performed by the rats. Following lesions, all animals exhibited approximately 6–11 ipsilateral rotations per min, which continued for the duration of the 90 min test (941.5 ± 22.5 net ipsilateral turn on average). In the absence of a transplant, the animals showed an increase in amphetamine-induced rotations over the subsequent tests (up to 25% increase by 18 weeks post lesion). In contrast, both groups of animals transplanted with VM tissue showed a significant reduction in rotational behavior (76% and 83% reduction after allogeneic and xenogeneic transplant respectively) compared to the sham treated group (Fig.2e; F_2,115_ = 49.51; Sidak’s post hoc test p < 0.0001). However, there was no difference between the groups grafted with allogeneic (rat) or xenogeneic (mouse) tissue (p = 0.646), or between the different treatments within each transplantation group (Fig.2e; treatment main effects F_3,106_ = 0.392; p = 0.759; graft and treatment interactions F_6,106_ = 0.718; p = 0.636). No graft-induced or amphetamine-induced post-graft dyskinesias were observed in any group.

Finally, the impact of a dopaminergic intra-striatal graft on motor function was assessed by counting the number of lateralized steps and vibrissae-elicit limb placement performed on the contralateral side to the lesion. No motor function recovery was observed in any of the groups, regardless of the type graft or treatment that the rats received (Table 1; vibrissae test F_6,106_ = 1.248; p = 0.288; stepping test backhand F_6,106_ = 0.285; p = 0.943; stepping test forehand F_6,106_ = 0.292; p = 0.939).

### 3.2 L-DOPA-induced motor dysfunctions

**Fig. 3:**
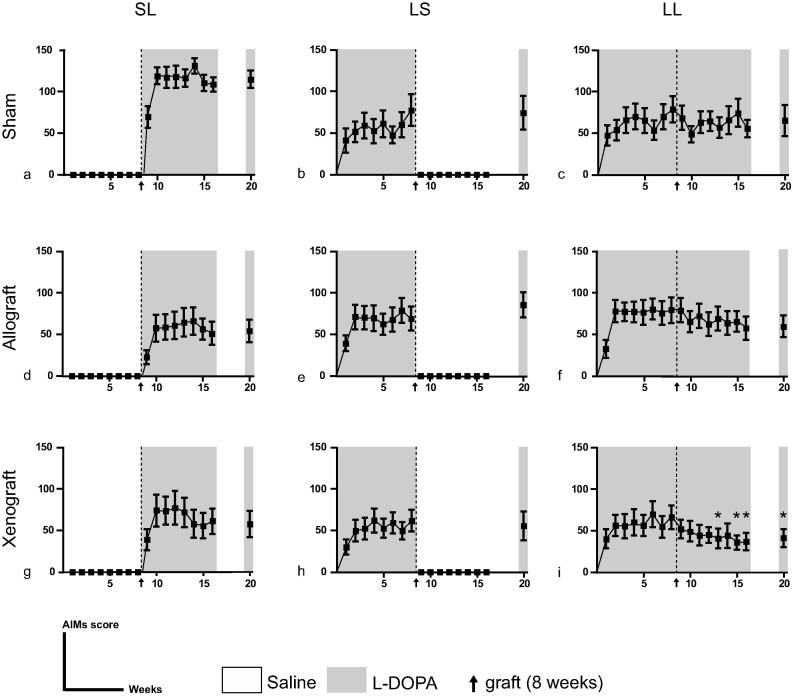
L-DOPA induced-dyskinesias. Average of abnormal involuntary movement (AIMs) scores rated before and after striatal transplantation (dotted line) of rat VM (middle panel) mouse VM (bottom panel) or sham surgery (top panel). The different treatments for each phase (I and II) are color-coded: white for saline or grey for L-DOPA. Data presented as mean ± SEM, ^*^p < 0.05 compare to last trial prior-transplant.

None of the saline treated control group (SS, in each transplantation set) displayed rotational behavior, beyond spontaneous ipsilateral rotations, which are commonly observed in 6-OHDA lesioned animals (Table 1, grey). As expected, they also failed to develop any AIMs. Since all animals obtained a consistent null score, the SS AIMs results are not presented and were excluded from statistical analyses. All L-DOPA treated groups (SL, LS, LL) progressively developed AIMs; from the first day of L-DOPA treatment (Fig. 3). Dyskinesia scores rapidly increased and reach a ceiling 2–3 weeks after initiation of the treatment in each group. Interestingly, delaying the onset of L-DOPA to phase II, in the sham transplantation group (SL), resulted in more severe dyskinesias than when the animals received the drug from phase I (Sham group: Fig.3a–c; SL: 116.5 ± 2.4, LS: 62.4 ± 2.1, LL: 60.1 ± 3.8). However, both allo- and xenogeneic fetal dopaminergic cells transplant, prior to the initiation of L-DOPA treatment in phase II, prevented exacerbation of AIMs (Fig.3d, g; Allograft SL: 54.4 ± 4.3; Xenograft SL: 62.9 ± 4.1). Rats receiving chronic treatment with L-DOPA throughout (before and after grafting, LL, Fig. 3c, f, and i) showed a decreasing trend in AIMs scores following VM transplantation. Nonetheless, this was only significant in the xenografted group, with a reduction of about 44% from week 14 (Repeated measures, F_16,176_ = 2.729; p = 0.0007).

L-DOPA-induced rotational behavior was recorded concomitantly. During the final L-DOPA challenge (12 weeks post-transplantation), the rats that have been grafted with fetal neurons showed a decreased in contralateral turns when compared to the sham surgery groups (Table 1; F_2,106_ = 11.35; p < 0.0001). No significant difference was found between the two types of graft (p = 0.226) or the 3 different regimes of L-DOPA administration (SL, LS, LL, main effect of Treatment: F_2,92_ = 1.718; p = 0.186).

Regarding stereotypic behavior, animals treated only with saline remained mostly inactive, as reflected by a mean stereotypic score under one (Table 1, SS, grey). All L-DOPA treated groups, however, exhibited similar stereotypic-like behaviors following L-DOPA administration, which increased over time. Neither the treatment regime, nor the type of graft had an impact on L-DOPA-induced stereotypies (week 20, Table 1, main effect of Treatment: F_3,106_ = 23.97, p < 0.001; of Graft: F_3,106_ = 0.64; p = 0.53).

### 3.3 L-DOPA treatment influences immune response

**Fig. 4:**
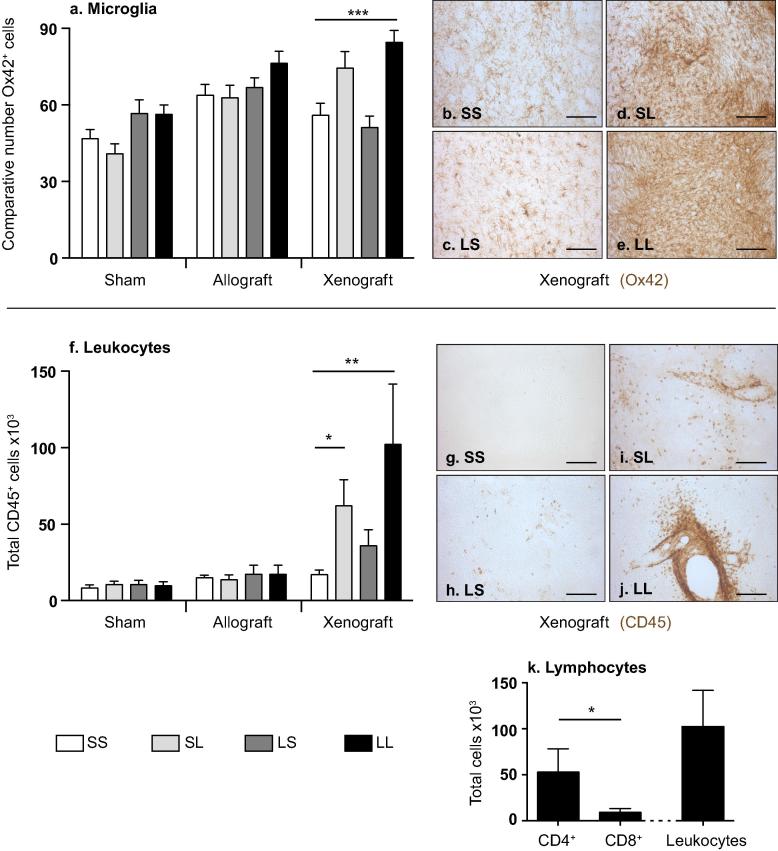
Immune response. (a) Comparative number of Ox42 positive cells observed around the graft. (b–e) Representative pictures of microglia immunochemistry staining (Ox42) of the xenografted area. (f) Total number of leukocytes in the grafted striatum and representative pictures of the xenografted striatum (g-j; CD45 staining). Scale bars: 100 μm. (a, f) Data are presented as mean ± SEM, n = 118, ^*^p < 0.05, ^**^p < 0.01,^***^p < 0.0001, relative to the corresponding SS group. (k) Average of CD4 and CD8 positive cells observed in the striatum of xenotransplanted animals, which received L-DOPA through both treatment phases. Presented as mean ± SEM, n = 12, ^*^p < 0.05.

Inflammation and immune response against the graft was evaluated using a microglia marker (Ox42) and a leukocyte marker (CD45). The presence of the graft (regardless of type), generally increased the number of microglia in the grafted area (Fig.4a–e, F_2,103_ = 9.89, p = 0.0002). An interaction was found between the type of graft (allogeneic or xenogeneic) and the treatment administered (F_6,103_ = 3.36, p = 0.005). Specifically, the xenograft significantly increased the number of Ox42^+^ cells in the transplanted area. Within the xenograft group, the most significant increase was found in the group treated with L-DOPA during both treatment phases (Fig.4e, LL) when compared to the other 3 treatment groups (Fig.4a; compared to SS and LS: p < 0.0001, SL: p = 0.02). Representative pictures of the microglia staining in the striatum of xenotransplanted animals are presented in Fig. 4(b–e).

The number of infiltrated leukocytes in the transplanted striatum was significantly higher in the xenogeneic graft group (Fig.4f, F_2,103_ = 8.89, p = 0.0003, post hoc analysis p < 0.001) but no difference was found between the sham and the allograft groups neither was there an effect of L-DOPA treatment in these groups (F_3,103_ = 1.81; p = 0.149). To better understand the effect of the different L-DOPA regimes on the xenograft group, this transplant group was analyzed separately. In the SS group of rats that had never been exposed to L-DOPA, very few CD45^+^ cells were observed (Fig.4g). In contrast, in all groups that had been treated with L-DOPA, a consistent increase in infiltrated cells was found throughout the striatum (Fig.4h-j). The mean number of leukocytes infiltrated in the 2 groups that received L-DOPA post-grafting was 3 and 6 times higher than in the SS control group (Fig.4f, χ^2^_3_ = 15.18, p = 0.0017). The most striking observation was the presence of agglomerated leukocytes along the blood vessels, close to the grafted area, which was seen in some of the rats treated with L-DOPA post-grafting (Fig.4i and j). Interestingly, in the same animals, the Ox42 staining revealed the presence of dense and aligned microglia, resembling gliosis (microglia scarring, Fig.4d and e).

In order to characterize the subtypes of T cells involved in the lymphocytic immune response, CD4 and CD8 immunostaining was performed on the brains of the rats grafted with xenogeneic tissue and treated with L-DOPA before and after grafting (Fig.4k, xenograft, LL). The number of CD4 positive cells was higher than the number of CD8 positive cells (Mann-Whitney test, U = 35, p < 0.033). Additional immunohistochemical analyses were performed on some animals in the xenotransplanted group to better characterize the type of immune response promoted by L-DOPA treatment post-transplantation (Supplementary Fig. 1). Intense astroglial (GFAP) activation was observed, as expected, with the additional presence of phagocytic cells (CD68+ cells) inside the graft and at the graft-host boundary. Some IL1β positive cells were identified located mainly within the graft itself, whilst the few cells positive for TNFα or IFNγ were found predominantly at the graft-host boundary.

To evaluate the effect of prolonged exposure to L-DOPA on the blood vessels and angiogenesis, sections were labeled immunohistochemically using a pan-vascular endothelium marker (RECA-1, data not shown). No significant difference was found between the contralateral (left) and ipsilateral (right) hemisphere to the graft (respectively 237.1 ± 12.3 μm^2^ and 233.2 ± 12.6 μm^2^ (F_1,40_ = 0.73, p = 0.397). Similarly, the treatment did not seem to have an impact on vascularization of the transplanted striatum (SS: 231.3 ± 11.8 μm^2^; LL: 234.8 ± 12.6 μm^2^). Since no difference was found between the SS and LL groups in the xenografted animals (considered to be the two most extreme scenarios), the other groups were not analyzed (F_1,40_ = 0.105, p = 0.748).

### 3.4 CSA does not interfere with AIMs development

**Fig. 5:**
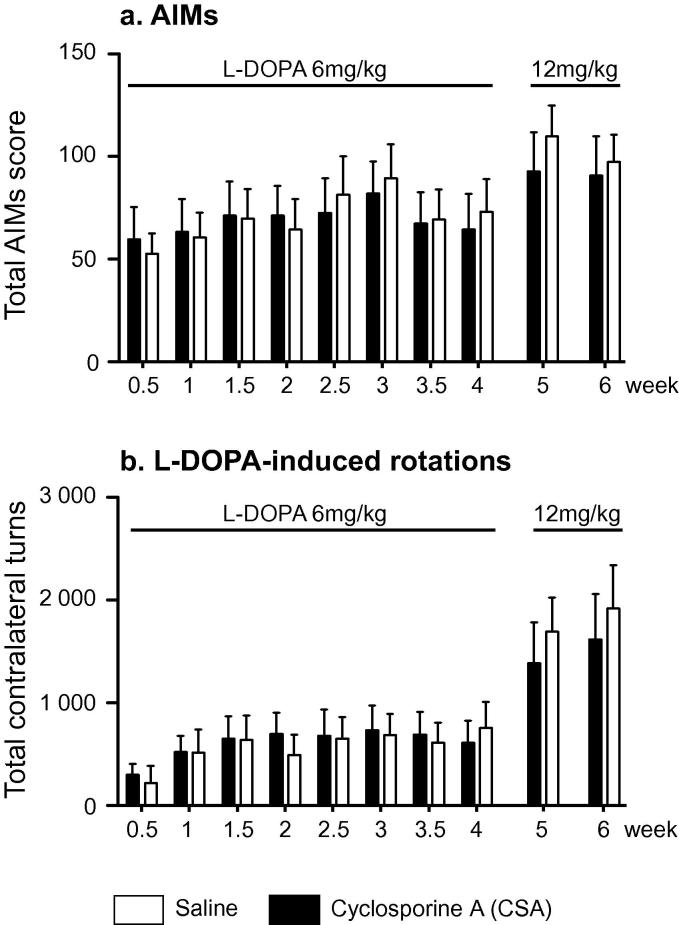
Chronic cyclosporine A treatment. There was no effect of CSA the development of L-DOPA induced dyskinesia (a) and rotational behavior (b).

In a separate experiment, L-DOPA (6 or 12 mg/kg) was administered concomitantly with CSA in order to assess potential interaction between the two treatments. As expected, both AIMs and contralateral rotations increased with L-DOPA dose (Fig. 5, AIMs: F_9,126_ = 9.46, p < 0.0001; rotations: F_9,126_ = 16.38, p < 0.0001). Animal receiving CSA developed similar level of L-DOPA-induced side effects when compared to animals treated with saline, regardless of the dose of L-DOPA (AIMs: F_1,126_ = 0.03, p = 0.875 and rotations: F_1,126_ = 0.01, p = 0.921). These data suggest that CSA treatment did not interfere with L-DOPA treatment.

## 4 Discussion

This study is the first systematic evaluation of the interaction between drug treatment of Parkinson’s disease, delivered before and/or after transplant, and the presence of a neuronal graft that is not immunologically compatible with the host. The results of this paper reiterate that VM allogeneic (rat) transplant (in absence of immunosuppression) and xenogeneic (mouse) transplant (in CSA treated animals) can survive well in the striatum of 6-OHDA-lesioned rats and produce functional effects, enabling a reduction of drug-induced motor asymmetry (Blunt et al., 1990, Gaudin et al., 1990, Blunt et al., 1992, Olsson et al., 1995, Lee et al., 2000, Carlsson et al., 2006, Steece-Collier et al., 2009, Tamburrino et al., 2015).

Overall, the beneficial effect of the transplantation in this experiment was limited and no improvement of motor functions, as assessed by stepping or whisker tests, was observed. This is likely a result of smaller grafts that were intentionally produced to enable a clear determination of the positive or negative effects of L-DOPA. Transplant studies in 6-OHDA rats commonly graft a higher number of cells (2–10 times more cells) (Dunnett et al., 1981, Nikkhah et al., 1994, Goren et al., 2005, Torres et al., 2007) and although it has been shown that transplantation of as little as 50,000 cells is enough to obtain reduction on the drug-induced rotation test, motor function recovery appears to require larger grafts (Bartlett et al., 2004). The low number of transplanted cells might also explains the fact that, contrary to what has been reported previously, the rats never developed post-transplant AIMs “off-drug” (during neither light or dark phases of the light cycle), or following amphetamine injection (Carlsson et al., 2006, Lane et al., 2006, Lane et al., 2009, Garcia et al., 2011). However, the grafts were large enough to impede the development of L-DOPA-induced dyskinesia, when L-DOPA was initiated post-transplantation. These finding are in line with Steece-Collier et al. (2009) study and lends itself in favor of intervening earlier in the disease course, a track which is being followed by the current EU funded clinical trial with fetal tissue transplantation TransEUro (Moore et al., 2014).

A key finding of this paper, that may have implications for long term graft survival and function, was that the presence of L-DOPA, specifically in the xenograft group, heightened the immunological response. The multifactorial approach used in this paper was validated by pathology very similar to that seen in post-mortem examinations of patients receiving graft material. We found increased microglial responses (Ox42+) and infiltrating white blood cells (CD45+) in the grafted area, including phagocytic cells (CD68+), in animals transplanted with xenogeneic VM and treated with L-DOPA post transplantation. Despite good graft survival and fibers outgrowth, the presence of IL1β positive cells, as well as TNFα and IFNγ in these animals, might hint toward a pro-inflammatory stage of the immune response, likely to be detrimental to the graft in the long run. Analysis of the brains of a patient with a well-surviving 18 months old VM transplant similarly reported the presence of immune cells invading, including phagocytic cells (CD68+) and lymphocytes T- and B-cells (Kordower et al., 1997). Three to four years post-transplant, patients who have received longer immunosuppressive treatment, showed a mild increase of GFAP or CD68 positive cells (mainly along the needle tracks) (Mendez et al., 2005). Interestingly, a patient transplanted bilaterally (16 and 12 years before his death) showed GFAP-immunoreactive astrogliosis on both side of the brain despite the presence of a good dopaminergic grafts, while another patient (at 22-years post-transplant), showed no sign of astrogliosis and had no remaining TH positive transplanted cells (Kurowska et al., 2011). All together, these data suggest that even though dopaminergic grafts seem to survive quite well in presence of infiltrating immune cells, at least for the first decade, they might eventually succumb to the host defense system. It is noteworthy that in a recent analysis of a patient’s brain 24 years after fetal transplant, very good survival of the dopaminergic cells was found with limited immune response (as assessed by IBA-1 and CD68 staining). Pertinent to this study, the patient did exceedingly well post-transplantation such that his medication steadily reduced and benefited from a complete withdrawal of L-DOPA two and half years after transplantation, while he remained under immunosuppression for few years after that (Li et al., 2016).

These observations of interactions between graft and drug treatment raise interesting clinical and biological questions of critical relevance to the future development of non-autologous cell transplantation for neurological conditions, and for the preclinical models that are used to promote that translational goal. The mechanism by which chronic L-DOPA treatment, administered post-transplantation, increases the immune response around non-compatible grafts remains unclear and but we have considered three hypotheses: 1) interference of L-DOPA with the action of CSA immunosuppressive treatment, 2) a direct action of L-DOPA on immune cell proliferation and/or activation, or 3) induction of blood-brain barrier impairment directly allowing an influx of immune cells. Competition for transport of L-DOPA and CSA through the intestinal wall and/or at the blood-brain barrier level might occur since CSA is transported through the P-glycoprotein 1, which can also provide a substrate for L-DOPA to enter both the intestinal lumen and the brain (Saeki et al., 1993, Vautier et al., 2008). Here we show that rats developed similar behavioral abnormalities (aka dyskinesia and rotational behavior) while exposed to L-DOPA, regardless of co-treatment with CSA, which implies that CSA does not interfere with L-DOPA penetration into the brain. However, we cannot rule out that L-DOPA might interfere with CSA transport. Lymphocytes are known to express TH, DOPA-decarboxylase, as well as DA receptors and transporters (Santambrogio et al., 1993, Ricci and Amenta, 1994, Ricci et al., 1998, Amenta et al., 1999, Ricci et al., 1999, McKenna et al., 2002, Cosentino et al., 2007) but even though a direct action of L-DOPA on immune cells at the periphery could be hypothesized, it is considered unlikely since L-DOPA is co-administered with a peripherally acting aromatic L-amino acid decarboxylase. Finally, L-DOPA treatment and dyskinesias have been associated with enhanced blood flow and blood vessel permeability in the striatum, which could facilitate access of peripheral immune cells such as leukocytes, to the brain (Westin et al., 2006, Lindgren et al., 2009, Ohlin et al., 2012). In this study we found no direct evidence of angiogenesis but we cannot exclude that modifications at the level of the blood brain barrier have occurred. Further work is required to establish to what extent each of these mechanisms plays a key role in the L-DOPA-induced increased host immune response phenomenon that we observed in xenogeneic transplanted animals.

Establishing a reliable animal model to study the parameters influencing the success of cell therapy for PD is a crucial but convoluted task. To the best of our knowledge we are the first group to attempt to replicate much of this multi factorial process. This has necessitated a thorough and systematic approach, which has produced some insights into a possible interaction between the graft, drug therapy and immune system. We have shown here that L-DOPA treatment, administered post-transplantation, can increase immune responses despite immunosuppressive treatment and believe that this should be taken into consideration for further trials of cell transplantation. With the upcoming development of stem cell-based therapies (Barker et al., 2015) the question remains as to whether the outcome would be the same with cells obtained from alternative sources and whether other common medications could influence other aspects of graft function and development.

## Conflict of interest

The authors declare no competing financial interests.

## Figures and Tables

**Table 1:** Behavioral tests (vibrissae and stepping) were conducted after lesion and again 10 weeks after grafting (week 18). Data are presented as Means ± SEM. One-way ANOVA analysis of variance comparing results after lesion and after grafting, all n.s. ^1^percentage of left limb responses to vibrissae stimulation, ^2^the number of steps performed with the left forelimb while moving the animal in the forehand or backhand direction. Stereotypy score and rotational behavior (week 20) were significantly lower in the saline groups (SS, grey) regardless of the type of transplant. No difference was found between the 3 other treatment groups treated with L-DOPA (SL, LS, LL).

Group		Vibrissae^1^		Stepping (backhand)^2^		Stepping (forehand)^2^		Stereotypic	L-DOPA induced
		Post lesion	Post graft	Post lesion	Post graft	Post lesion	Post graft	Behavior	Rotations
Sham	SS	15.0 ± 2.2	9.3 ± 4.9	7.7 ± 1.8	8.5 ± 1.5	2.7 ± 0.7	3.3 ± 0.9	0.33 ± 0.33	−28.79 ± 8.84
	SL	15.3 ± 5.2	14.9 ± 4.6	9.8 ± 1.4	8.1 ± 0.9	3.0 ± 1.2	2.1 ± 1.2	11.00 ± 0.65	497.1 ± 96.03
	LS	14.1 ± 3.7	9.1 ± 3.3	8.6 ± 1.9	9.1 ± 1.7	3.3 ± 1.5	1.4 ± 0.3	11.12 ± 1.65	432.96 ± 195.70
	LL	17.5 ± 4.9	14.5 ± 5.1	9.1 ± 1.6	5.5 ± 1.2	2.4 ± 0.6	1.9 ± 0.7	9.62 ± 1.85	470.7 ± 132.40

Allograft	SS	14.4 ± 2.9	15.4 ± 5.6	8.1 ± 0.9	9.3 ± 1.1	2.7 ± 0.4	2.6 ± 0.4	0.40 ± 0.40	−38.53 ± 17.95
	SL	14.2 ± 2.5	13.8 ± 4.1	7.4 ± 0.6	7.6 ± 1.4	2.3 ± 0.8	1.5 ± 0.6	8.45 ± 1.89	126.9 ± 50.53
	LS	16.8 ± 3.2	16.7 ± 6.9	8.7 ± 1.3	9.4 ± 1.1	2.5 ± 0.6	1.5 ± 0.3	7.91 ± 1.29	264.6 ± 111.6
	LL	14.5 ± 2.9	21.7 ± 6.1	8.5 ± 1.0	8.6 ± 1.1	3.3 ± 0.6	2.8 ± 0.4	10.91 ± 1.51	251.9 ± 70.82

Xenograft	SS	14.6 ± 3.2	26.2 ± 7.1	7.9 ± 1.1	6.2 ± 1.4	2.6 ± 0.5	1.7 ± 0.9	0.40 ± 0.27	−22.45 ± 6.78
	SL	14.9 ± 3.1	23.6 ± 8.0	8.0 ± 1.0	8.1 ± 1.0	2.5 ± 0.6	2.5 ± 0.6	7.70 ± 1.93	31.78 ± 20.66
	LS	14.3 ± 3.7	24.4 ± 6.4	8.4 ± 1.6	4.7 ± 1.1	2.8 ± 1.0	2.2 ± 0.6	9.09 ± 1.81	119 ± 42.94
	LL	16.1 ± 3.5	19.4 ± 6.4	8.7 ± 1.2	5.5 ± 1.3	3.2 ± 0.6	2.2 ± 0.5	10.08 ± 1.77	107.9 ± 46.39

## Appendix A Supplementary data

Supplementary data 1 Characterization of the immune response observed in xenotransplanted animals treated with L-DOPA both pre- and post-transplantation using immunohistochemistry. (Scale = 100 μm).
