# Supplementary material for: Influence of chronic L-DOPA treatment on immune response following allogeneic and xenogeneic graft in a rat model of Parkinson’s disease
Source: Brain Behav Immun. 2017 Mar;61:155–64. doi: 10.1016/j.bbi.2016.11.014 (PMC5325122; doi:10.1016/j.bbi.2016.11.014)
Supplement: Supplementary data 1 — Characterization of the immune response observed in xenotransplanted animals treated with L-DOPA both pre- and post-transplantation using immunohistochemistry. (Scale = 100 μm). [file mmc1.docx]

Supplementary figure 1: Characterization of the immune response observed in xenotransplanted animals treated with L-Dopa both pre- and post- transplantation using immunohistochemistry. (Scale=100μm).
